# Agreeable Smellers and Sensitive Neurotics – Correlations among Personality Traits and Sensory Thresholds

**DOI:** 10.1371/journal.pone.0018701

**Published:** 2011-04-27

**Authors:** Ilona Croy, Maria Springborn, Jörn Lötsch, Amy N. B. Johnston, Thomas Hummel

**Affiliations:** 1 Smell and Taste Clinic, Department of Otorhinolaryngology, University of Dresden Medical School, Dresden, Germany; 2 Department of Psychosomatic Medicine, University of Dresden Medical School, Dresden, Germany; 3 Pharmazentrum Frankfurt/Zentrum für Arzneimittelforschung, Entwicklung und Sicherheit, Institute of Clinical Pharmacology, Goethe-University, Frankfurt am Main, Germany; 4 Clinical Neuroscience, Eskitis Institute of Cell and Molecular Therapies, Griffith University, Nathan, Australia; Duke University, United States of America

## Abstract

Correlations between personality traits and a wide range of sensory thresholds were examined. Participants (N = 124) completed a personality inventory (NEO-FFI) and underwent assessment of olfactory, trigeminal, tactile and gustatory detection thresholds, as well as examination of trigeminal and tactile pain thresholds. Significantly enhanced odor sensitivity in socially agreeable people, significantly enhanced trigeminal sensitivity in neurotic subjects, and a tendency for enhanced pain tolerance in highly conscientious participants was revealed. It is postulated that varied sensory processing may influence an individual's perception of the environment; particularly their perception of socially relevant or potentially dangerous stimuli and thus, varied with personality.

## Introduction

Personality research often explores the development of and influences on personality traits, characteristics that are sometimes defined as “enduring tendencies or habitual patterns of behavior, thought, and emotion” [1]. Many models of personality, exploring and defining specific traits have been developed, some more complex than others. The five-factor model of personality [2] enables a description of human personality in a relatively economical way. It is intended to supply a comprehensive taxonomy of traits using only five basic categories- extraversion, neuroticism, agreeableness, openness and conscientiousness and thus provides opportunity to effectively explore potential influences on the development of personality traits and their relationships with other parameters such as sensory ability.

Indeed, one of the most interesting areas in current personality research deals with the problem of how personality may be shaped during development. What induces people to differ from each other in the way they think, feel or behave in certain situations? The last few decades of personality research and clinical praxis have seen the emergence of nature-nurture-interaction hypotheses in answer to such fundamental developmental issues [3], [4]. These suggest that basic tendencies are constitutionally predisposed but are also developed and shaped during experiences with and within the environment. Thus, the environment enables subtle shaping of apparently underlying genetic components of personality.

Prior to this study almost no research has explored the influence of variation in sensory thresholds on individual differences in personality, even though research has demonstrated that there are significant individual differences in visual, auditory, olfactory and gustatory capacity (e.g. [5], [6], [7]) as well as in tolerance to pain ([8], [9], [10]. Thus, this study set out to systematically examine whether there maybe relationships between sensory thresholds and inter-individual personality differences.

Sensory ‘constitution’ could be an individual variable associated with and helping to form personality characteristics. This hypothesis is seated in the notion that people do not have an objective picture of the world surrounding them, but rather, a person-specific filtered one. The varying capacities of peoples' sensory systems would form one part of a possible ‘sensory-filter’ system applied by all to their perception of their environment. Such a stable, rigid filter could profoundly influence an individual's perception of the world and therefore influence their thoughts, behavior and emotions relative to their environment. In addition, the attention given by an individual to a particular stimuli or to a particular form of sensory input could also shape such a ‘sensory-filter’. Attention to particular kinds of sensory stimuli could be modulated by many factors including by current emotional state. For example, people suffering from posttraumatic stress disorder (PTSD) have been shown to process unpleasant, potential harming, stimuli in a preferred way [11], [12].

‘Thought’ experiments can be used to graphically illustrate this ‘sensory-filter’ concept. If one lived with increased strong feelings of pain, one might perceive the world as a rather unpleasant place. A major behavioral motivation for such a person would include reduction of pain, so that harm avoidance would become highly important and even prevalent. Indeed there is evidence suggesting that neuroticism is related to pain perception ([9], see below).

Another straightforward example to follow is that of the influence of blindness on personality. Ammerman and colleagues (1986) provided evidence demonstrating that blind adolescents tended to be more dependent on others, more introverted and to exhibit enhanced anxiety than sighted adolescents. This might be a very appropriate adjustment to the disease, ensuring that they develop and use reliance on others to help them safely navigate their environment [13]. The current experiments explore the hypothesis that not only the total loss of one of the senses could and should induce personality change, but that intra-individual variability occurring in every sensory system, and leading to intra-individual differences in sensory capacity for each sense, may also influence personality.

Goldman and colleagues explored a very similar hypothesis when they attempted to correlate auditory threshold in 42 student participants with measurements of sensation seeking [14]. They reported that sensation seekers were significantly less sensitive to auditory thresholds than the normative population; supporting the notion that there is an influence of the capacity of the auditory system on personality. Unfortunately, this study used ascending instead of random choice thresholds of testing, so the influence of decision-making processes on auditory threshold remains somewhat unclear. However, even when mindful of this proviso, their interpretation of their results was that people with low auditory sensitivity might be in search of stimulation, supporting a possible role of sensory-filtering in externalization of personality traits.

When considering the processing steps that determine how people perceive the environment, it seems plausible that capacity for sensory processing could influence the ‘picture of the world’, people build and thus, the development of patterned behavioral responses to ‘that world’. Relationships between sensory systems and personality might play out through personality development as discussed above. Alternatively, the capacities of sensory systems and instigation of personality traits may share the same underlying genetic origins. In any case, looking at ways personality traits might relate to sensory variables is a valuable first step in addressing such questions.

The aim of the present study was to systematically explore coherence between a wide range of sensory thresholds and personality traits. The study used the NEO-FFI to measure personality dimensions. It is based on a five-factor-model of personality model, a widely accepted and often validated theory for description of personality [2], [15]. Concomitant measurement of sensory thresholds focused on assessment of olfactory, trigeminal chemosensory, electrical and gustatory detection thresholds, as well as establishing individual's trigeminal chemosensory and electrical pain thresholds. The decision to attempt correlation of these senses with personality was primarily driven by the wealth of experience in our laboratory with detailed and precise determination of chemosensory perception. To ensure the reader's familiar with these senses, they are discussed in some detail below.

Chemosensory perception including olfaction, gustation and trigeminal perception are included amongst the evolutionarily ‘oldest’ senses. Environmental cues, typically processed through the **olfactory channel** are often associated with perception of food, but ‘danger’ and ‘social’ stimuli can also be communicated using olfaction [16]. Olfaction also seems to play a key role in providing information about the emotional state of others [17], [18]. In a recent experiment Prehn-Kirstensen and colleges presented anxiety-induced and sport-condition sweat samples to 28 participants and collected fMRI data during presentation. They showed that the ‘anxiety’ sweat was processed in areas related to empathy, while the ‘sport condition’ sweat was not [17]. Given the social communication function of the olfactory system, one might expect personality traits, related to social relationships, to be influenced by the effective sensitivity of olfactory system. We could therefore postulate that agreeableness, a trait strongly related to social skills, would be positively correlated with olfactory sensitivity.

**Gustatory perception** is also activated during eating, so that potentially dangerous nature of the food (often associated with the bitterness of the food), and also the nutrition value of food (sweetness) can be estimated. It is not immediately clear how an enhanced or reduced ability to perceive food (oral) stimuli –within a normal range – would influence personality. There is evidence, at least in very young children, that presentation of sweet stimuli can act as a form of analgesia, reducing the negativity and stress associated with medical procedures [19]. Thus, to get a broad overview of different senses, their variability and potential interrelationship of personality, gustation was also explored.

Receptors of the **trigeminal chemosensory channel** also lie within the nasal cavity. Stimulation of this system in a healthy person leads to perception of a burning/stinging sensation. This typically occurs while eating spicy foods, but also during the detection of potential dangers, such as fires.

Finally, stimuli processed through **electrical cutaneous channels** includes touch and tactile information, but also information about potential risks such as chemical and/or mechanical damage of the skin. Thus, we also analyzed **pain perception** by presenting increasing stimuli to the trigeminal and electrical cutaneous channels. For both, the trigeminal chemosensory as well as the electrical cutaneous channels we could hypothesize enhanced neuroticism in people with high sensitivity to potentially painful and/or dangerous stimuli, both conceptually and also on the basis of some, albeit limited, evidence.

There are sporadic studies dealing with sensory sensitivity of the systems described above and personality. In one study, pain tolerance to cold water was examined in 56 Japanese students and correlated with their performance in the Maudsley Neuroticism and Extraversion Scales and in the Manifest Anxiety Scale [10]. Results indicated a significant coherence between pain sensitivity and high neuroticism and anxiety values, and with low extraversion values [10].

In a recent study, Paine and colleagues induced visceral and somatic pain in 18 healthy volunteers by distension of an oesophageal balloon or application of nail-bed pressure, respectively. During the ‘pain application’, measurements of cardiovascular variables including blood pressure and also skin conductance were obtained. Additionally participants were asked to complete the ‘Big-Five’ Inventory. Significant coherence between enhanced neuroticsm and decreased extraversion was recorded, with significant, pain-related, cardiac vagal tone slope change. The authors interpreted this result in the context of development of functional visceral pain syndrome, which is overrepresented in highly-neurotic patients [9].

Generally, research exploring sensory detection thresholds and their relationship with personality is relatively rare. Besides the connection between sensation seeking and auditory threshold, described in the study of Goldman and colleagues above [14], we could find only two studies dealing with personality and taste thresholds. In one study Zverev and Mipando (2008) obtained taste detection thresholds in 60 volunteers and correlated these with the results from application of the Eysencks Personality Inventory. They found no coherence between taste sensitivity and determined personality traits [20]. In another study published in 1967 Corlis and colleges compared the personality of students with high and low quinine taste sensitivity [21]. They found quinine-sensitive participants to be more “intuitive” than the insensitive tasters.

In an older study, 140 students were asked to complete a questionnaire scoring personality on the intraversion-extraversion scale and to performing a test examining olfactory sensitivity [22]. In this study a small, but significant, correlation (r = 0.23) between extraversion and enhanced olfactory sensitivity was reported. In another publication reporting two studies with a rather small sample sizes of 12, respectivly 26 participants, neuroticism, but not extraversion was found to be related with enhanced olfactory sensitivity to some, but not to all of the analysed odors [23]. An intreguing study from Zhou and colleagues explored olfaction and emotional abilities [18]. They asked 22 pairs of female roommates to identify the body odor of their roommate from one of three t-shirts. The higher the women scored on a self-rating questionnaire measuring emotional awareness, the better they were able to perform the task, implying a potential link between emotional awareness and olfactory discrimination threshold.

Thus, the evidence linking personality and chemosensory sensitivity is mixed – particularly as there is little evidence linking sensitivity in one chemosensory modality with that of another chemosensory modality [24]. The current study was developed to explore multi-modal chemosensory abilities in people and potential relationships between specific chemosensory modalities and personality traits.

## Methods

### Ethics Statement

The study followed the Declaration of Helsinki on Biomedical Research Involving Human Subjects and was approved by the Ethics Committee from the University of Dresden Medical School. All participants provided written informed consent.

### Participants

A total of 124 healthy subjects (41 men, 85 women, aged 18 to 52 years, mean = 24 years; standard deviation = 5) participated in this study; most of whom were graduate students or members of the Technical University of Dresden Medical School. Completion of a detailed medical history form by each participant enabled confirmation of their good physical health. Demographic data from the participants is shown in Table 1. Data from these participants has previously analyzed with respect to correlations between the different sensory system [24], but not with respect to individual differences in personality.

**Table 1:** Participant's demographic data.

		female					male				
		N	Mean	SD	Min	Max	N	Mean	SD	Min	Max
age		85	24	5	19	52	41	25	5	18	48
Personality dimensions (T-Scores)	Neuroticism	85	48	9	32	70	41	46	9	31	75
	Extraversion	85	51	8	25	70	41	49	11	27	70
	Openess	85	48	11	26	71	41	49	9	27	74
	Agreeableness	85	53	9	29	72	41	54	9	31	74
	Conscientiousness	85	54	9	34	80	41	55	8	34	75

Data of the NEO-FFI is provided converted to t-scores, provided by the German normative sample described in the manual. T-scores shown are standardized scores with a mean of 50 and a standard deviation of 10.

### Materials and Procedure

Following the taking of a detailed medical history, participants were asked to complete the German form of the NEO-FFI questionnaire [25], [26]. Subsequent assessment of sensory thresholds was conducted with the testing sequence counterbalanced across all participants. Participants were given breaks of 5 to 10 min between the various tests.

#### NEO-FFI Questionnaire

Personality was assessed using the reliable and validated NEO-FFI questionnaire based on the ‘BIG FIVE’ theory of personality. The self-rating questionnaire consists of 60 different statements which prompt responses on a 5-point Likert-scale varying from “total disagreement” to “total agreement”. A sum score for each of the five personality dimensions “neuroticism”, “extraversion”, “openness for new experiences”, “agreeableness” and “conscientiousness” is used to build a personality profile. Average time for completion of the questionnaire is 10 minutes.

#### Threshold testing

To prevent visual cues from prompting responses during chemosensory measurements, participants were obliged to wear an eye mask during testing. **Detection thresholds** were obtained using a three-alternative, forced-choice, modified staircase method of stimulus presentation (see below). The thresholds assessed were: trigeminal chemosensory (CO_2_), olfactory (PEA – phenyl ethyl alcohol), gustatory for sour (citric acid) and salty stimuli (NaCl), and cutaneouos electrical stimuli. **Pain thresholds** were obtained for cutaneous electrical and trigeminal chemosensory stimuli. Beginning with the detection threshold, stimulus intensity was increased (linearly) upwards to the point when the participant indicated that the stimulus became noticeably painful. This procedure was repeated at least once. If the second estimate differed from the first by more than one step, the procedure was repeated a third time. An average of the two estimates (or, where three measurements had been taken, the average of the three estimates) was used as the final measure.

#### Trigeminal chemosensory thresholds

A short gaseous CO_2_ stimulus was delivered via an olfactometer (Olfactometer OM2S, Burghart Instruments, Wedel, Germany) to the participant's nasal mucosa (duration 500 ms, rise time <20 ms; total flow 6 l/min). Measurements started at a concentration of 3% v/v CO_2_. Concentrations were increased stepwise by 3% CO_2_ up to a concentration of 30% v/v, thereafter concentration steps of 5% CO_2_ were used. An interval of approximately 15–20 s was provided between each presentation of individual stimuli.

For **detection threshold**, a three-alternative forced-choice task and a staircase paradigm starting at 3% CO_2_ concentration were used. One CO_2_ stimulus and two blanks (room air) were presented at each dilution step. Subjects were asked to indicate whether they perceived a stimulus. Two successive correct identifications or one incorrect identification triggered a reversal of the staircase. Detection thresholds were estimated as the mean of the final four out of seven staircase reversals. After assessment of the detection threshold, each participant's **pain threshold** was determined. CO_2_ concentrations were increased until the participant indicated that the stimulus became painful. This procedure was repeated at least once. If the second estimate differed from the first estimate by more than one concentration step, the procedure was repeated once more. An average of the two estimates (or, in case where three measurements had been taken, the average of the three estimates) was used as the determined pain threshold.

#### Electrical thresholds

Electrical thresholds were obtained using a constant voltage device (PowerLab 26T; ADInstruments, Spechbach, Germany). Stimuli were applied with a stimulating bar electrode, placed at the forehead. Shock intensity was increased from 0 to 20 mA in steps of 0.1 mA.

For determination of **detection thresholds** a 3-alternative forced choice paradigm was used, similar to that described above. Subjects received three stimuli per trial (two with 0 mA, one with electrical stimulation; stimulus duration 2 ms; interstimulus interval between the triplet of stimuli: 2–4 s; interstimulus interval between triplets: approximately 15–20 s). Two successive correct identifications of the impulse or one incorrect identification triggered a reversal of the staircase. Tactile detection was represented by the average voltage determined by the mean of the last four out of seven staircase reversals. After assessment of the detection threshold, **pain threshold** was obtained. The electrical stimuli were increased stepwise up to the point when the participant indicated that the impulse was perceived as painful. This procedure was replicated at least once. If the second estimate differed from the first estimate by more than one intensity step, the procedure was repeated once more. An average of the two or three estimates obtained was used to describe pain threshold.

#### Olfactory threshold

Olfactory detection threshold was assessed birhinally with the “Sniffin' Sticks” test (Burghart Instruments, Wedel, Germany) [27], [28]. In this validated test odors are presented in felt-tip pens. For odor presentation, one pen at a time – with the cap removed - is placed directly in front of the nostrils at a distance of approximately 1 to 2 cm (for a detailed description of the test procedures please see [27]). Odor thresholds were obtained for the rose-like odor phenyl ethyl alcohol (PEA) presented in 16 1∶2 dilution steps starting from a 4% solution. PEA is commonly used for olfactory threshold testing and correlations between PEA thresholds and Butanol thresholds are acceptable high [29].

Using a three-alternative forced-choice task and a staircase paradigm starting at low PEA concentrations, one pen with the odorant and two blanks were presented at each dilution step. Again, two successive correct identifications or one incorrect identification triggered a reversal of the staircase. Odor detection threshold was represented by the mean of the last four out of seven staircase reversals.

#### Gustatory thresholds

Gustatory detection thresholds were assessed for sour (citric acid) and salty (NaCl) stimuli. Administration of the taste stimuli was based on the principles used with the “Taste strips” [30] where 1 cm^2^ of filter paper is impregnated with a tastant. The dried filter papers are then applied to the tongue. In the current test, based on extensive previous experience with the taste strips [31], the strips applied to the tongue were impregnated with 14 dilutions each of salty and sour stimuli, starting from lowest concentrations of 0.3 g/ml citric acid and 0.25 g/ml NaCl Dilutions were made in geometric series of 1∶3 with water as the solvent. Using a ‘whole-mouth’ paradigm, participants received 3 strips with only one containing tastant at a given concentration. Their task was to describe whether the strip had a ‘taste’ or not. After each stimulus presentation, the participants rinsed their mouth with fresh tap water. Where participant's response was correct 3 times in a row, the dilution step was noted down as gustatory threshold.

## Results

Data was analyzed using the SPSS 17 Software (SPSS Inc., Chicago, Ill., USA). For comparison of our sample with the German normative sample [25] raw data of the NEO-FFI personality dimensions were converted into T-scores and then analyzed using single t-test.

Pearson's correlation analysis between the personality dimensions and the assessed sensory thresholds from the whole sample was performed. Bonferroni-Holm- corrections for multiple comparisons adjusted for dependent measurements have been performed for all correlations (k = 10) [32]. To clarify the independent contribution of the sensory capacity measurements to the personality trait values, linear multiple regression analysis was also performed.

### Personality traits

Results from the questionnaire are provided in Table 1. Participants scored significantly lower on the neuroticism scores (*p* = 0.005) and significantly higher on the agreeableness (*p*<0.001) and on the conscientious-scale (*p*<0.001) than the German normative sample [25]. This is probably related to the skewed (University) population who made up the test sample.

### Correlation between sensory thresholds and personality traits

**Figure 1:**
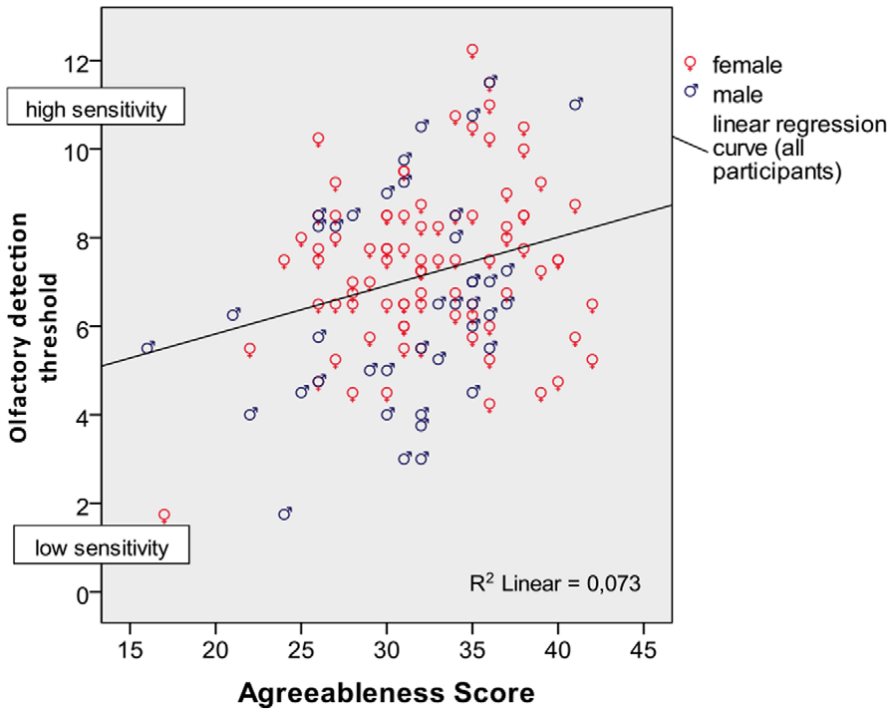
Odor detection threshold and agreeableness scores in male and female participants.

**Figure 2:**
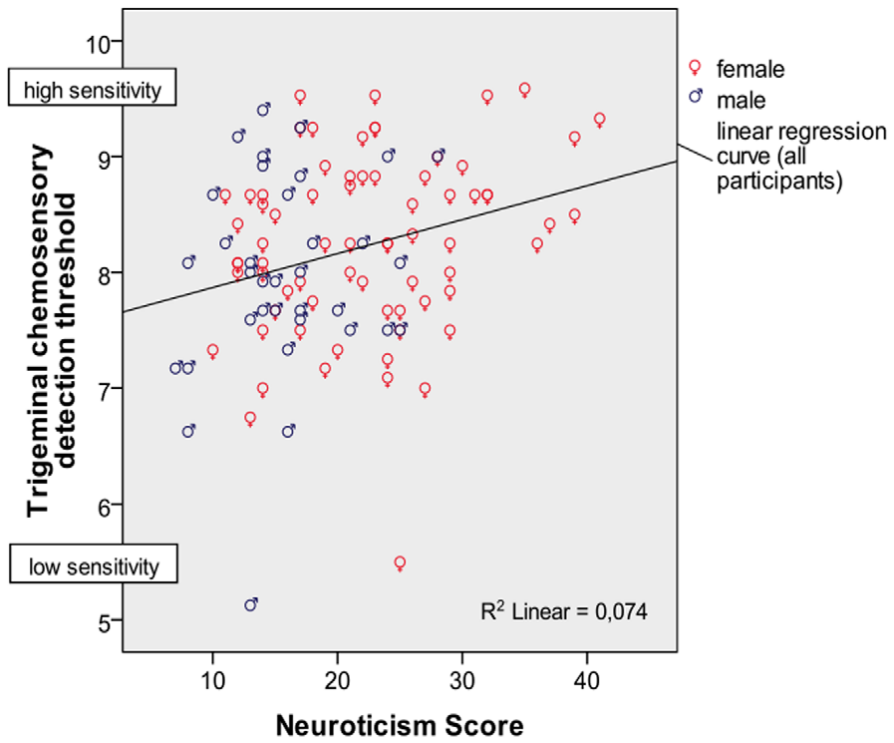
Trigeminal chemosensory detection threshold and neuroticism scores in male and female participants.

Correlations between sensory thresholds and personality traits are presented in Table 2. A small, but significant positive coherence was observed between **agreeableness** and **odor detection sensitivity** (*r* = 0.269, *p_corrected_* = 0.02, see Figure 1). A small, but significant positive correlation was evident between **neuroticism and trigeminal chemosensory detection sensitivity** (*r* = 0.272, *p_corrected_* = 0.05, see Figure 2). A positive correlation was also evident between assessed neuroticism and both pain thresholds (trigeminal: *r* = 0.225 *p_uncorr_* = 0.021; electrical cutaneous: *r* = 0.184, *p_uncorr_* = 0.042), but these correlations did not survive corrections for multiple measurements. Without correcting for multiple measurements, there were significant negative correlations between conscientiousness and trigeminal chemosensory perception sensitivity (*r* = −0.213, *p_uncorr_* = 0.28) and with electrical cutaneous pain sensitivity (*r* = −0.181, *p_uncorr_* = 0.46), but both correlations vanished on correction for multiple measurements.

**Table 2:** Pearson's correlation coefficients between personality traits and sensory detection and pain thresholds.

Sensory Thresholds		Personality traits				
		Neuroticism	Extraversion	Openness to experience	Agreeableness	Conscient-iousness
olfactoryN = 126	Detection	−.026	.100	.064	.269^**^	.007
		n.s.	.n.s.	n.s.	**.002 (0.02)**	n.s.
GustatoryN = 126	Detection salty	.065	.022	.077	.005	−.122
		n.s.	n.s.	n.s.	n.s.	n.s
	Detection sour	.114	−.108	−.049	.022	−.015
		n.s.	n.s.	n.s.	n.s.	n.s
Trigeminal chemosensoryN = 106	Detection	.272^**^	−.085	.003	.024	−.213^*^
		**.005 (0.05)**	.387	.972	.803	**.** ***028 (n.s.)***
	pain	**.** 225^*^	n.s.	n.s.	n.s.	n.s.
		**.** ***021 (n.s.)***	n.s.	n.s.	n.s.	n.s.
Electrical cutaneousN = 123	Detection	.176	−.051	−.059	.032	−.058
		n.s.	n.s.	n.s.	n.s.	n.s
	pain	**.** 184^*^	.153	.088	.125	−.181^*^
		**.** ***042 (n.s.)***	n.s.	n.s.	n.s.	**.** ***046 (n.s.)***

*Note.* In each line first correlation coefficients are presented, followed by level of significance. If p<0.05 the level of significance corrected for multiple comparison is presented in brackets. Correction was performed with the Bonferroni-Holm-method adjusted for dependent measurements (k = 10) [32]. n.s. … not significant. k … correction coefficient.

No significant coherence was observed between the value of the extraversion and openness personality traits in the NEO-FFI questionnaire and any of the threshold tests. For the gustatory channel no significant correlation with the value of the personality traits was observed.

### Sensory predictors of the personality traits

Multiple regression analysis revealed two models that significantly predict agreeableness (see Table 3). Model one included only the olfactory detection thresholds (p = 0.046), while model two included olfactory detection thresholds and trigeminal chemosensory sensitivity (p = 0.04). For trigeminal chemosensory only the pain threshold, not the detection threshold, contributes significantly to the model. Two significant models could be used to predict neuroticism values (see Table 4). Model one included trigeminal chemosensory sensitivity (p = 0.022), in which only detection threshold, not pain threshold, contributes significantly. Model two also contains electrical cutaneous sensitivity (p = 0.02), however, neither of these variables alone significantly directs outcome.

**Table 3:** Predictors of Agreeableness.

	NEO-FFI Agreeableness			
	Model 1		Model 2	
Variable	*B*	95%CI	*B*	95%CI
Constant	29,34**	[26.12, 32.16]	31.74**	[27.56, 35.93]
Olfactory detection	0.45*	[0.01–0.89]	0.47*	[0.027–0.90]
Trigeminal chemosensory detection			0.04	[−0.08, 0.16]
Trigeminal chemosensory pain			−0.07*	[−0.13, −0.003]
*R^2^*	0.04		0.08	
*F*	4.08*		2.88*	
*ΔR^2^*			0.04	
*ΔF*			2.23	

Only the inclusion of the variables olfactory detection, trigeminal chemosensory detection and pain led to significant regression models, therefore only those models are presented.

*Note.* N = 103. CI = confidence interval.

*p<0.05.

**p>0.01.

**Table 4:** Predictors of Neuroticism.

	NEO-FFI Neuroticism					
	Model 1		Model 2		Model 3	
Variable	*B*	95%CI	*B*	95%CI	*B*	95%CI
Constant	19.28**	[13.83, 24.72]	24.48**	[18.69, 32.27]	27.24**	[20.59, 35.05]
Olfactory detection	0.17	[−0.57–0.91]	0.23	[−0.48, 0.94]	0.112	[−0.602, 0.826]
Trigeminal chemosensory detection			−0.20*	[−0.40, 0.003]	−0.20*	[−0.40, −0.003]
Trigeminal chemosensory pain			−0.05	[−0.17, −0.04]	−0.06	[−0.13, 0.09]
Electrical cutaneous detection					−6.65	[−14.8, 1.52]
Electrical cutaneous pain					−0.18	[−0.54, 0.17]
*R^2^*	0.00		0.09		0.13	
*F*	0.21		3.37*		2.82*	
*ΔR^2^*			0.09		0.03	
*ΔF*			4.94		1.91	

Only the inclusion of the variables olfactory detection, trigeminal chemosensory and electrical cutaneous detection and pain led to significant regression models and thus only those models are shown.

*Note.* N = 103. CI = confidence interval.

*p<0.05.

**p>0.01.

No significant model of the sensory variables predicting the personality traits conscientiousness, extraversion and openness was determined.

## Discussion

The main result of this study, confirming our initial hypotheses, was that there is an apparent relationship between certain personality traits and sensory capacities. This finding supports the notion that sensory capacity may provide a filter through which we perceive the world, and that this filter may influence the picture we receive of the world. Interestingly the various chemosensory systems seem to be of differing importance in helping to influence personality traits. We found no coherence between personality traits and gustatory modality (mainly related to eating) but significant coherence between personality traits and olfactory, trigeminal sensory and electrical cutaneous modality; systems usually thought to be related to detection of social cues and awareness of danger. This concurs with results of a recent study that also found no coherence between gustatory sensitivity and personality [20]. It seems reasonable that sensory systems processing environmental cues such as social relationships and potential danger should more strongly influence personality shaping than systems that processing cues related to eating. Moreover, when analyzing the data describing the different thresholds we tested for each individual, we found no overall correlation between these thresholds. So it seems that there is no such thing as a “generally sensitive” person, but rather that people differ quite widely in the sensitiveness of their specific chemosensory modalities [24].

We interpret the correlation between **enhanced odor sensitivity and agreeableness** to indicate that high olfactory sensitivity might mirror increased interest in social matters including social odors. Multiple regression analysis additionally revealed one significant model predicting agreeableness values with olfactory detection and trigeminal chemosensory sensitivity. As we did not find a clear significant correlation between agreeableness and trigeminal chemosensory pain sensitivity, we rather suspect a possible statistical suppression effect here.

Agreeable people can also be described as “cooperative, considerate, empathic, generous and kind” people, and thus this personality trait indicates the ability to form congenial social relationships with others [4]. As odors have the potential to communicate information about the emotional state of others [17], an enhanced ability to detect such odors could support empathy and social awareness in recipients. The recent study showing female roommates who are better able to identify their roommate also score higher in assessments of emotional competence, further supports this hypothesis [18].

Wider exploration of sex differences in emotional and in olfactory competence shows similar patterns of gender response. Women do not only have generally higher agreeableness scores [33], but also typically outperform men in odor processing abilities [34]. Interestingly, previous research showed coherence between effective social function and olfactory performance in people with autism. In fact, autistic children with especially high social impairment had the lowest levels of olfactory perception [35].

**Trigeminal chemosensory detection sensitivity** has been found to be related to enhanced neuroticism. Environmental cues that are typically processed using this modality are strongly related to ‘danger’ signals. In contrast to olfactory or gustatory stimulation, trigeminal stimulation is described as becoming painful through to becoming unbearable at high concentrations. While the data described herein, indicating enhanced trigeminal and electrical pain sensitivity occurring in conjunction with higher levels neuroticism did not survive correction for multiple measurements, it nevertheless led in the very same direction. Use of a greater number of participants drawn from wider social groupings may have enabled this relationship to withstand detailed statistical comparison. Other studies have also reported relationships between pain sensitivity and evident neuroticism [9], [10]. Neuroticism is a trait “that encompasses the tendency to experience the world as distressing or threatening” [4]. Based on our results we would argue that participants who are very sensitive to potentially dangerous and inherently painful trigeminal stimuli maybe more likely to interpret the world as an unpleasant one, which would result in enhanced neuroticism scores. Although this explanation would seem eminently plausible, correlation analyses do not allow causal interpretation. It would be equally plausible to explore this pattern the other way around: That is, people who score high on “neuroticism” may tend to subjectively anticipate experiences as negative or potentially damaging [36], and could, therefore, be more likely to detect potentially unpleasant stimuli. Results concurring with this hypothesis have been described in a subgroup of people with pathologically negative emotional expression – women with post-traumatic stress disorder [11]. Significantly negative correlations were described between women with enhanced PTSD scores and event-related potentials evoked in response to odors and also the trigeminal stimulant CO2. Peak latencies for CO_2_ and for a very unpleasant odor were reduced in the population showing more extreme PTSD-related responses, indicating a preferred processing of unpleasant stimuli in those patients.

A rather surprising coherence between the ‘**conscientious**’ trait and reduced trigeminal chemosensory sensitivity was evident in these results, as well as a correlation between the ‘**conscientious**’ trait and **reduced pain sensitivity** in the trigeminal chemosensory. The same tendency was evident between the ‘**conscientious**’ trait and electrical cutaneous sensitivity (see table 2). Neither significant correlation, nor the apparent tendency, was evident following correction for multiple measurements, so cautious interpretation of these results must be applied. However, both pain sensitivity measurements showed the same tendency for enhanced pain tolerance in more conscientious participants, and thus it warrants some consideration. Pain thresholds were assessed using an ascending scale until the participant indicated the stimulus as painful. This is in contrast to detection thresholds which were assessed in a multiple choice way, and thus, are relatively difficult to influence. Moreover, a motivational component is very likely to modulate the outcome of pain threshold measurements. Therefore, the apparently enhanced pain tolerance in highly conscientious participants maybe associated with the enhanced motivation these people have to perform a task ‘well’. Desire for compliance with study results and a motivation to ‘please’ and do ‘the right thing’ must be carefully considered when dealing with the personality trait conscientiousness and its impact on pain perception.

When comparing sensory capacity with personality, there are several potential biases which must be carefully considered. Firstly, neither sensory capacity nor personality are observed directly. To test these sensory capacities psychophysiological measurements were used which, as careful one collects and examines them, maybe biased by tiredness and inattention. Also, mood during sensory testing may influence results, especially when pain thresholds are tested [37], Given that pain threshold assessment is conducted using an ascending series, absolute threshold maybe biased by alterations in decision making processes. On the other hand psychophysiological testing seemed more appropriate than other methods of measuring sensory processing, because it might reflect the best the subjective experience of the participant. In further studies a control for affective state and tiredness should be included. Moreover, personality traits were assessed via a self-rating-questionnaire. The NEO-FFI is widely used and has proved to have a good reliability and validity [25], but nevertheless processes like perceived social desirability might play a role in the responses a person provides.

It is also important to consider the theoretical background of the personality trait description used in this study. The NEO-FFI is based on the five-factor model of personality [26], [38], which was generated using a lexical approach to verbs describing personality. The five-factor structure was found to be quite stable and concordant with other models of personality [39], however one of the major critiques of this approach is that lexical words might not represent all facets of human personality equally, but rather might represent oversimplified descriptors of human personality. Another critique of this factor modeling of personality, which is especial important here, is based around the possibility of a biological basis of such factors. The factors are constructions of relatively stable variables describing personality, but the biological basis for such grouping has been questioned [40]. One attempt to establish a biological basis for the ‘Big five’ was attempted by Jang and colleagues. They claim to have found two genetic factors underlying each of the five personality traits [41]. Looking for coherence between personality and sensory capacity with a more biological basis and a more detailed personality inventory, might enhance the probability of revealing relationships between genetic inheritance and personality trait.

A significant limitation of this study is the relatively homogeneous sample it used. Participants were young and healthy medical students or members from the Technical University of Dresden. Thus the population was highly educated, high-socioeconomic group with little or no chronic disease or debilitation. Thus, a bias towards higher instances and certainly higher valuing of certain personality traits such as enhanced conscientiousness or agreeableness and reduced neuroticism might be anticipated. Comparison of the data from these participants with that from a German normative population sample clearly showed this sample bias. Variation within the expression of traits explored was relatively small in this sample, again suggesting homogeneity within it. As correlations normally downsize, when homogeneous samples are analyzed [42], it maybe that the coherence between personality traits and sensory capacity described here augments analyzing a broader spectrum of personality values. Nevertheless it is not possible, at this stage, to generalize these results to a wider population. Future studies should encompass a sample that better represents the wider ‘normal’ population.

**In conclusion**, to our knowledge, this is the first systematic study correlating thresholds in different sensory systems and certain personality traits. We focused on olfactory, gustatory, trigeminal chemosensory and electrical cutaneous thresholds, covering a range of processed environmental cues related to social relationships, eating and detection of potential danger. The study showed coherence between capacities of the olfactory system and agreeableness, possibly moderated through enhanced social perception abilities. Additionally, enhanced sensitivity in the sensory systems detecting danger was found to be related to high neuroticism. It could be that the sensitivity with which one is able to perceive environmentally important stimuli influences perception of the environment and is therefore is able to influence development and expression of personality traits.
